# Inflammatory myofibroblastic tumors in children: a single-center retrospective study of clinical features, management, and outcomes

**DOI:** 10.3389/fped.2025.1667711

**Published:** 2025-09-04

**Authors:** FeiYue Guo, QiangQiang Cui, Yang Lei, HongWei Xi

**Affiliations:** ^1^ Department of General Surgery, Shanxi Provincial Children’s Hospital, Taiyuan, China; ^2^ Department of Pathology, Shanxi Provincial Children’s Hospital, Taiyuan, China

**Keywords:** inflammatory myofibroblastic tumor, pediatrics, diagnosis and treatment, surgery, ALK-targeted therapy

## Abstract

**Background:**

Inflammatory myofibroblastic tumor (IMT) is a rare mesenchymal neoplasm of intermediate malignant potential. While its clinicopathologic features have been described in adults, comprehensive data in the pediatric population remain limited.

**Methods:**

We retrospectively reviewed pediatric patients diagnosed with IMT at Shanxi Provincial Children's Hospital between January 2016 and June 2024. Clinical data, imaging, histopathology, immunohistochemistry (IHC), and outcomes were systematically analyzed.

**Results:**

A total of 16 patients with complete clinical data and follow-up information were included. There were 10 male and 6 female patients, with a median age of 5.0 years. The primary tumor locations and clinical manifestations were diverse: 8 cases were located in the abdominal and pelvic cavities, presenting with abdominal pain, nausea, vomiting, and fever; 4 cases were located in the head, neck, and facial region, with 3 presenting as painless localized masses and 1 with inspiratory dyspnea; 1 case each occurred in the chest wall, gluteal region, and left upper limb, all presenting with painless localized masses; and 1 case occurred in the lung, presenting with cough, sputum, and recurrent respiratory infections. All 16 patients underwent surgery; three developed local recurrence requiring re-operation. At last follow-up, 15 were disease-free and one remained stable on ALK-targeted therapy.

**Conclusion:**

Pediatric IMT is a rare, low-grade malignancy with favorable prognosis. Complete surgical resection remains the cornerstone of treatment. ALK-targeted therapy may benefit patients with unresectable or recurrent disease. Long-term surveillance is warranted due to the risk of recurrence.

## 1 Introduction

Inflammatory myofibroblastic tumor (IMT) is a rare mesenchymal neoplasm of intermediate malignant potential, predominantly affecting children and adolescents (1). Histologically, it is defined by a proliferation of myofibroblastic spindle cells admixed with a variable inflammatory infiltrate. The 2020 WHO classification of soft-tissue tumors recognizes IMT as an intermediate-grade lesion with a propensity for local recurrence and, rarely, distant metastasis (2). IMTs can affect multiple sites throughout the body, with pulmonary involvement being more common in adults, while extra-pulmonary IMT is more frequently seen in younger patients (3). In children, IMT most frequently involves the abdomen and pelvis, followed by the head and neck, and thoracic regions. Additionally, there have been reported cases involving the genitourinary tract, peripheral soft tissues, bones, and the central nervous system (4–6). The clinical manifestations of pediatric IMT are highly variable, primarily depending on the size and location of the tumor. The absence of specific symptoms, signs, and typical systemic or imaging features makes it challenging to distinguish IMT from other tumors clinically, leading to frequent misdiagnoses prior to surgery (7, 8).

Owing to the low incidence of pediatric IMT, the existing body of research predominantly comprises case reports or small-sample studies, with comprehensive investigations into its clinicopathologic characteristics, treatment strategies, and prognosis remaining relatively sparse. This study retrospectively analyzed the clinical data of 16 pediatric IMT patients at our hospital, aiming to summarize the clinical and pathological features, treatment methods, and prognosis, and to provide a deeper understanding of this rare disease in children, helping guide its standardized diagnosis and treatment in clinical practice.

## 2 Materials and methods

### 2.1 Study design and patient selection

This was a single-center, retrospective cohort study conducted at Shanxi Provincial Children's Hospital. We screened the pathology registry for all patients aged 0–18 years who were diagnosed with inflammatory myofibroblastic tumor (IMT) between January 2016 and June 2024. Inclusion criteria were: 1. histopathologically confirmed IMT on surgical or biopsy specimens; 2. complete medical records, including baseline demographics, clinical presentation, imaging, treatment details, and follow-up data; 3. at least 12 months of post-operative follow-up or until death. Patients with inadequate tissue samples or lost to follow-up within the first year were excluded. Ultimately, 16 patients met the inclusion criteria. This study was conducted in full accordance with the Declaration of Helsinki. The protocol was reviewed and approved by the Ethics Committee of Shanxi Provincial Children's Hospital (Approval No:IRB-WZ-2025-031), and written informed consent was obtained from each participant's parent or legal guardian before any data were collected.

### 2.2 Histopathological diagnosis

Hematoxylin-eosin (H&E) stained sections were reviewed independently by two pediatric pathologists blinded to clinical data. Diagnoses were established according to the 2020 WHO Classification of Tumors of Soft Tissue and Bone. Immunohistochemistry (IHC) was performed on 4 µm formalin-fixed paraffin-embedded sections using a standard avidin-biotin peroxidase method. In cases where diagnosis is challenging, advanced auxiliary techniques such as fluorescence *in situ* hybridization (FISH) and genetic testing were employed to confirm the diagnosis.

### 2.3 Treatment strategies

All patients were discussed by a multidisciplinary team (MDT) comprising pediatric oncologists, surgeons, pathologists, and radiation oncologists. Treatment decisions were individualized based on tumor resectability, anatomical location, local invasion, metastatic status, and patient comorbidities. Surgical resection with negative margins (R0) was the preferred modality whenever feasible. Adjuvant chemotherapy or targeted therapy with ALK inhibitors was considered for unresectable, recurrent, or metastatic disease. Radiotherapy was reserved for refractory or margin-positive cases after MDT consensus.

### 2.4 Follow-up

During the first year after surgery, children were required to visit our hospital's outpatient department every 3 months for follow-up examinations, which included imaging and laboratory examinations. Starting from the second year, follow-up intervals were extended to every 6 months. For children who were unable to visit the outpatient department on schedule, the research team conducted follow-up via telephone to ensure the continuity and completeness of the follow-up process.

## 3 Results

### 3.1 Clinical characteristics

Sixteen pediatric patients (10 males, 6 females) met the inclusion criteria and were included in the study. Median age at diagnosis was 5.0 years (range 4 months-15 years). Tumor location determined the clinical presentation, which was otherwise nonspecific. The abdomen/pelvis was the most common primary site (8/16, 50.0%); these patients chiefly reported abdominal pain, nausea, vomiting, and low-grade fever. Tumors of the head, face, or neck accounted for four cases (4/16, 25.0%): three presented as painless, progressively enlarging masses (two forehead, one right cervical), whereas the subglottic lesion caused inspiratory stridor. Solitary masses were also observed on the chest wall (*n* = 1), gluteal region (*n* = 1), and left upper arm (*n* = 1). One pulmonary lesion manifested with persistent cough, productive sputum, and recurrent respiratory tract infections. After confirming the diagnosis of IMT, all patients underwent whole body examinations, and no distant metastases were detected. Detailed clinical data of the pediatrics patients are summarized in Table 1.

**Table 1:** Clinical characteristics, management, and outcomes of 16 pediatric IMT patients.

No	Location	Sex/Age	Clinical manifestation	Positive laboratory result	Treatment	Recurrence/metastasis	Treatment after recurrence	Follow-up Time	Status at last follow-up
1	Left upper arm	F/5 Year	Painless mass	—	CR	No	—	16 Months	DFS
2	Chest wall	M/14 Month	Painless mass	—	CR	No	—	12 Months	DFS
3	Forehead	M/5 Year	Painless mass	—	CR	No	—	17 Months	DFS
4	Right neck	M/7 Year	Painless mass	—	CR	No	—	36 Months	DFS
5	Gluteal region	M/14 Year	Painless mass	—	CR	No	—	51 Months	DFS
6	Abdominopelvic cavity	F/15 Year	Intermittent abdominal pain	Hb 90 g/L, ESR 24 mm/h	CR	No	—	54 Months	DFS
7	Abdominopelvic cavity	F/10 Year	Abdominal bloating and pain, accompanied by vomiting	WBC16.5 × 10^9^/L, Hb 102 g/L, CRP50.47 mg/L	CR	No	—	36 Months	DFS
8	Lung	M/4 Year	Cough, sputum, and recurrent respiratory infections	WBC19 × 10^9^/L, Hb 96 g/L, CRP 80.75 mg/L, ESR 84 mm/h	CR	No	—	46 Months	DFS
9	Forehead	M/5 Year	Painless mass	—	CR	No	—	20 Months	DFS
10	Abdominopelvic cavity	M/4 Year	Intermittent abdominal pain	Hb 105 g/L	CR	Yes	CR	54 Months	DFS
11	Abdominopelvic cavity	M/9 Year	Intermittent abdominal pain and fever	WBC15.2 × 10^9^/L, Hb 94 g/L, CRP 110.05 mg/L, ESR76 mm/h	CR	Yes	Palliative resection + ALK inhibitors	46 Months	Survival with tumor
12	Abdominopelvic cavity	F/6 Month	Abdominal bloating and pain, accompanied by vomiting and fever	WBC21 × 10^9^/L, Hb87 g/L, ESR 105 mm/h	CR	No	—	30 Months	DFS
13	Abdominopelvic cavity	F/4 Month	Abdominal bloating and pain, accompanied by vomiting and fever	WBC24 × 10^9^/L, CRP33.80 mg/L, ESR 90 mm/h	CR	No	—	36 Months	DFS
14	Abdominopelvic cavity	M/4 Year	Intermittent abdominal pain	WBC17.5 × 10^9^/L, Hb98 g/L, ESR 22 mm/h	CR	No	—	108 Months	DFS
15	Abdominopelvic cavity	M/3 Year	Intermittent abdominal pain	WBC20 × 10^9^/L, Hb95 g/L	CR	No	—	88 Months	DFS
16	Subglottic	F/7 Year	Inspiratory dyspnea	Hb101 g/L, CRP30.20 mg/L	R1 resection	Yes	CR	48 Months	DFS

F, female; M, male; WBC, white blood cell count; Hb, hemoglobin; CRP, C-reactive protein; ESR, erythrocyte sedimentation rate; CR, complete resection; DFS, disease-free survival.

### 3.2 Laboratory findings

Systematic laboratory evaluations were available for all 16 patients. Leukocytosis was documented in 7 children (43.8%), whereas 9 (56.3%) presented with anemia. Among the 12 patients in whom C-reactive protein (CRP) was measured, 5 (41.7%) exhibited elevated levels. Erythrocyte sedimentation rate (ESR) was determined in 10 children, and 6 (60.0%) had values above the age-specific reference range. Hepatic and renal function tests, coagulation profiles, and a panel of serum tumor markers were within normal limits in every case.

### 3.3 Imaging findings

All 16 patients underwent at least one imaging examination; eight (50.0%) were evaluated with two or more complementary modalities. Ultrasound (US) was performed in 13 cases (81.3%), computed tomography (CT) in nine (56.3%), magnetic resonance imaging (MRI) in two (12.5%), and flexible laryngoscopy in one (6.3%).

#### 3.3.1 Ultrasound

Of the 13 US examinations, seven targeted the abdomen. Three of these displayed heterogeneous, predominantly hypoechoic masses exhibiting concentric-layer (“target”) patterns in transverse scans and “sleeve-like” configurations longitudinally. Two additional cases revealed solid, hypoechoic abdominal masses with irregular internal echoes and no evidence of liquefaction or calcification; Color-Doppler flow imaging (CDFI) demonstrated scant intralesional vascularity. In two further abdominal cases, US showed circumferential bowel-wall thickening with luminal gas and focal dilatation, accompanied by hyperechoic foci and ascites.

**Figure 1:**
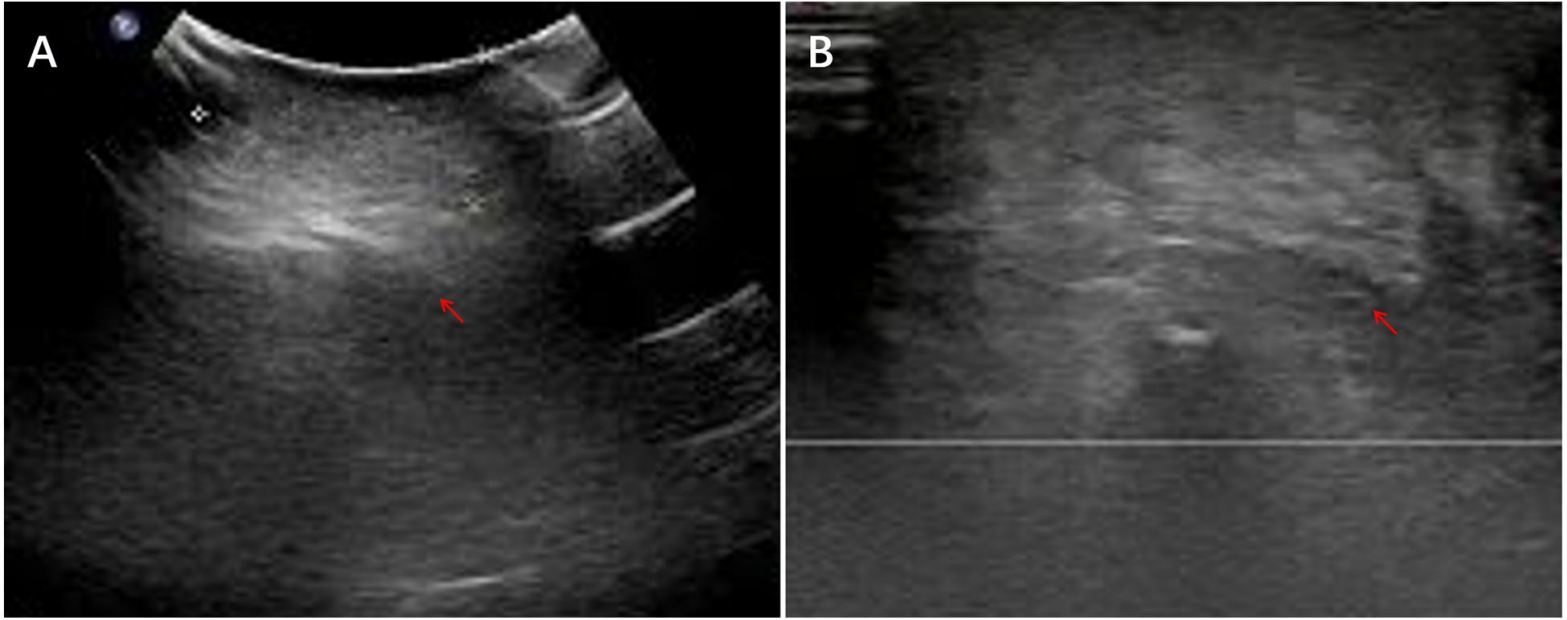
Ultrasound images of the left gluteal region IMT. **(A)** A hypoechoic solid subcutaneous mass with heterogeneous internal echotexture. **(B)** The mass shows poorly defined margins and an irregular morphology.

Extraperitoneal tumors (left upper arm, chest wall, right neck, gluteal region; *n* = 4 each) were uniformly solid and hypoechoic with well-defined margins and minimal CDFI signals. Two forehead lesions presented as cystic–solid masses with irregular, poorly circumscribed borders and mildly heterogeneous internal echoes. Representative ultrasound images of the gluteal region IMT are shown in Figure 1.

#### 3.3.2 Computed tomography

Nine patients underwent CT; four received intravenous contrast. Most lesions exhibited indistinct margins and heterogeneous attenuation, with punctate or patchy calcifications and foci of cystic change.

Pelvic IMT (*n* = 1): A bulky pelvic soft-tissue mass containing multiple nodular and patchy calcifications was identified. The lesion abutted adjacent small-bowel loops without clear planes, and post-contrast imaging revealed no appreciable enhancement.

Right pelvic IMT (*n* = 1): A mildly hyperattenuating mass measuring 1.6 cm × 2.9 cm × 2.5 cm displayed mild-to-moderate, heterogeneous enhancement, within which a small central cystic component was noted. The mass compressed the bladder and showed indistinct borders with the external iliac vein and adjacent small intestine.

Ileocecal IMT (*n* = 1): CT revealed concentric mural thickening of the ileum, most pronounced at the jejunal transition, accompanied by irregular nodular projections and loss of the adjacent fat-plane. The cecum and terminal ileum showed marked, asymmetric wall thickening, and post-contrast images demonstrated heterogeneous enhancement throughout the affected segments.

**Figure 2:**
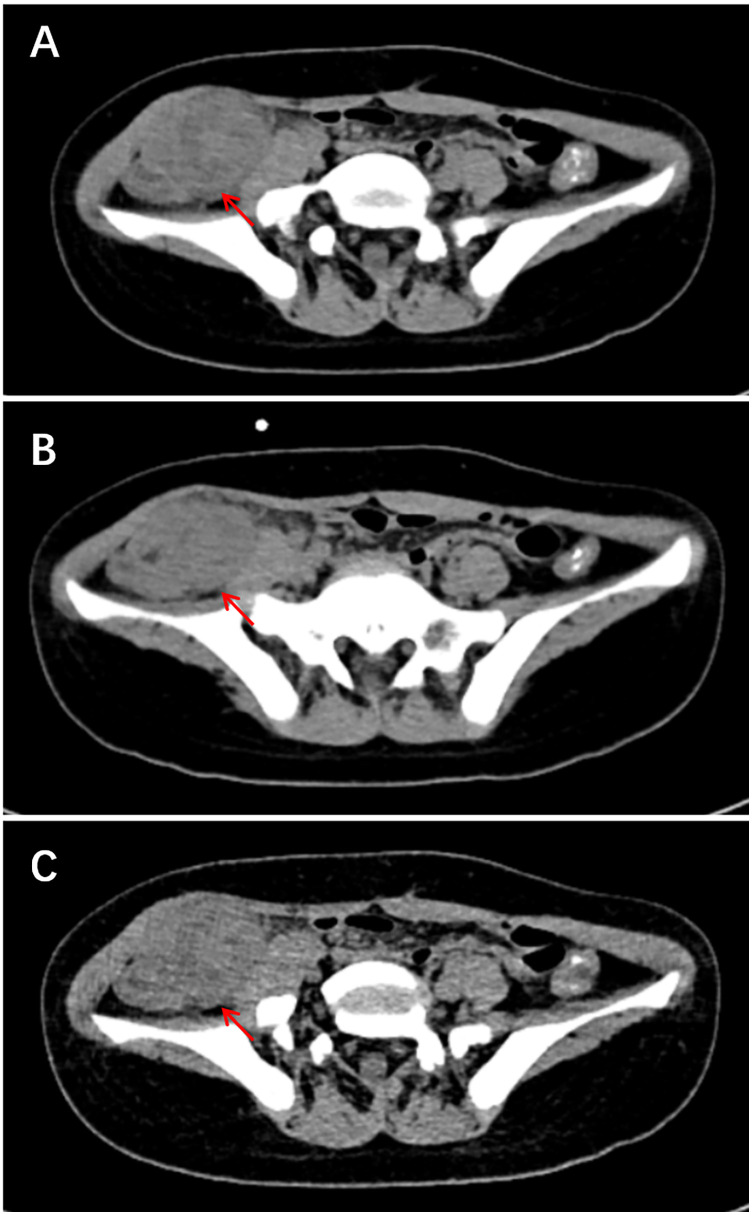
Ct images of right pelvic IMT. **(A)** CT image shows focal clustering of bowel loops in the right iliac fossa with an associated heterogeneous mass. **(B–C)** Coronal and sagittal reconstructions reveal the lesion measures approximately 4.1 cm × 3.9 cm and is accompanied by blurring of the surrounding fat planes.

Subglottic IMT (*n* = 1): CT demonstrated a polypoid, hyperdense mass arising from the right posterior tracheal wall at the level of the thyroid cartilage. The lesion measured 1.2 cm × 0.9 cm × 2.8 cm and protruded intraluminally, resulting in approximately 70% luminal narrowing. Post-contrast images revealed marked, homogeneous enhancement throughout the lesion. CT images of right pelvic IMT are presented in Figure 2.

#### 3.3.3 Magnetic resonance imaging

##### 3.3.3.1 Two patients underwent MRI

Right cervical IMT (*n* = 1): On T1-weighted images the lesion exhibited subtle, ill-defined isointensity within the paravertebral musculature and intermuscular fascial planes at C4-T1; corresponding T2-weighted images demonstrated mildly hyperintense signal with indistinct margins, compatible with a fibro-inflammatory process.

**Figure 3:**
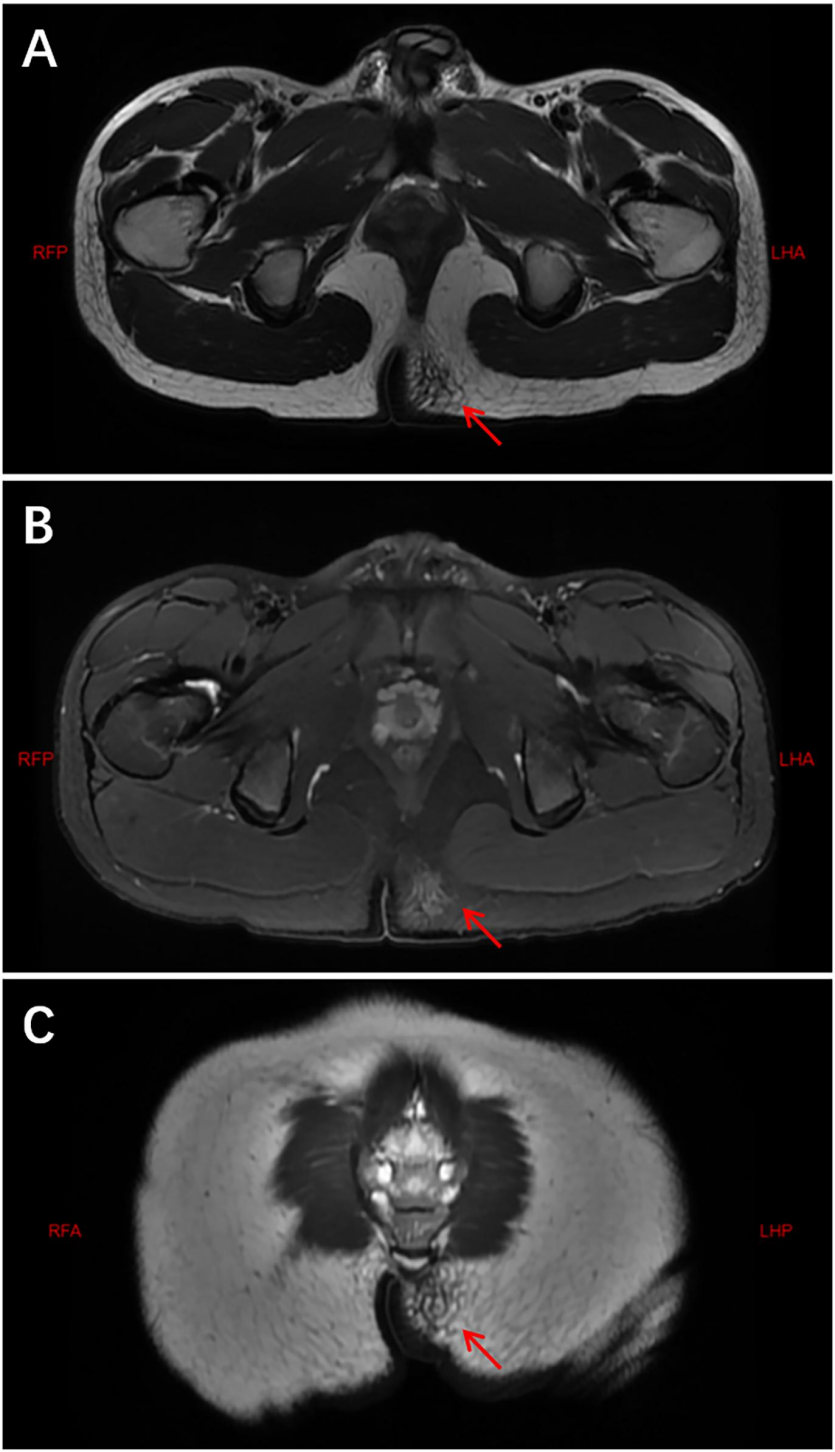
MRI findings of the left gluteal lesion. **(A)** T1-weighted image reveals a slightly hypointense mass within the subcutaneous fat. **(B)** The mass appears mildly hyperintense on T2-weighted imaging. **(C)** Fat-suppressed sequence demonstrates a hyperintense lesion with ill-defined margins, measuring approximately 3.0 cm × 2.3 cm × 1.9 cm.

Gluteal IMT (*n* = 1): An irregular, slightly elongated nodular lesion was identified in the subcutaneous fat, showing isointensity on T1-weighted images, mild hyperintensity on T2-weighted images, and subtle hyperintensity on fat-suppressed sequences with indistinct margins. The MRI manifestations of the left gluteal region IMT are presented in Figure 3.

#### 3.3.4 Endoscopy

Flexible laryngoscopy disclosed a 1.0 cm firm, irregular neoplasm arising from the posterior tracheal wall at the subglottic level; the lesion displayed a rough mucosal surface and reduced the tracheal lumen by approximately 70%.

### 3.4 Pathological findings

All 16 pediatric patients received a definitive diagnosis based on comprehensive histopathological evaluation of surgically resected specimens.

Macroscopically, the tumors exhibited heterogeneous appearances: they were round, oval, or irregularly lobulated masses with gray-red to gray-white cut surfaces, firm consistency, and a solid architecture. Larger lesions frequently displayed cystic change or a gelatinous texture; intra-abdominal tumors often exhibited adhesions to adjacent structures.

Histologically, all lesions were composed of bland, spindle-shaped myofibroblasts arranged in variably cellular fascicles admixed with a polymorphous inflammatory infiltrate rich in plasma cells, lymphocytes, and scattered eosinophils. Mitotic activity was low and necrosis was absent.

IHC staining showed the following: ALK(+) in 10 cases (10/16), Vimentin(+) in 11 cases (11/14), SMA(+) in 12 cases (12/15), Desmin(+) in 7 cases (7/11), CD117(−) in 10 cases (10/11), S-100(−) in all 16 cases (16/16), Dog-1(−) in 8 cases (8/8). The Ki-67 proliferation index ranged from 2% to 35%.

**Figure 4:**
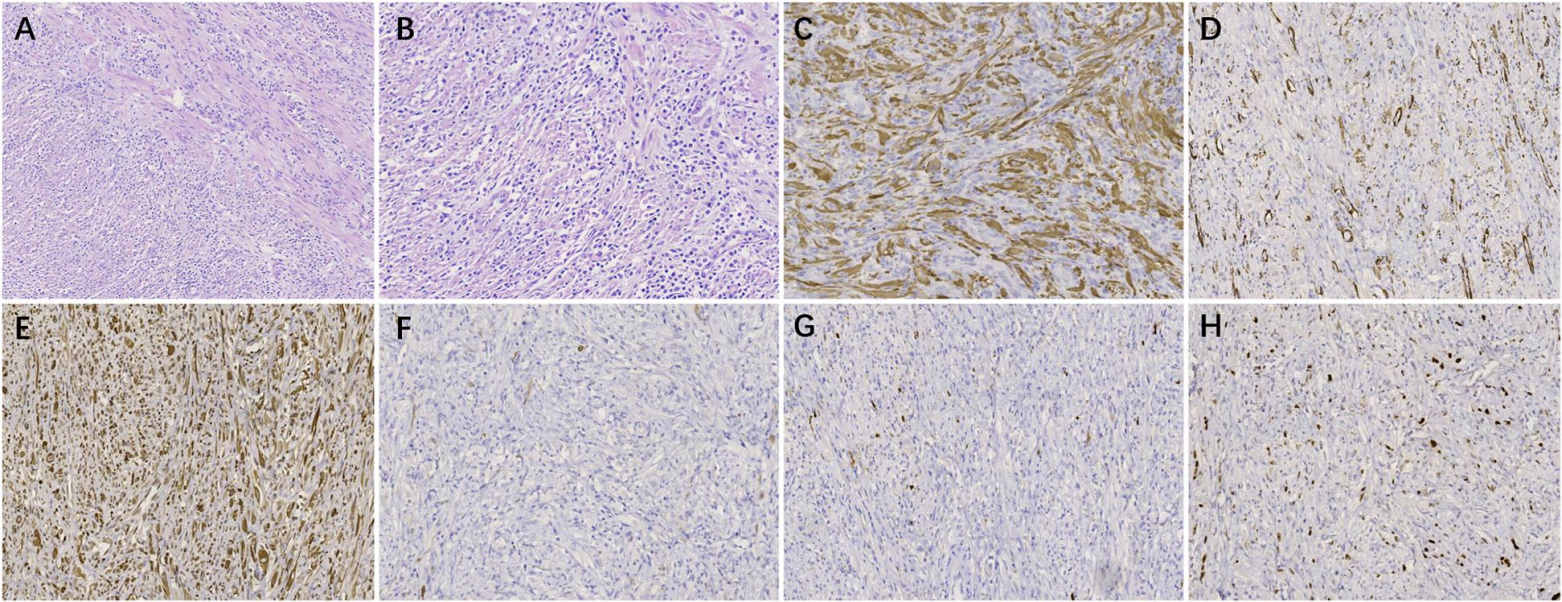
The pathological examination of mesenteric IMT. **(A)** HE staining (×100). **(B)** HE staining (×200). **(C)** IHC staining for ALK (+) (×200). **(D)** IHC staining for SMA (+) (×200). **(E)** IHC staining for Desmin (+) (×200). **(F)** IHC staining for CD117 (–) (×200). **(G)** IHC staining for S-100 (–) (×200). **(H)** IHC staining for Ki-67, approximately10% (×200).

FISH for ALK rearrangement was performed in three cases; two demonstrated ALK gene rearrangement, whereas one was negative. Detailed IHC results are shown in Table 2. Typical HE staining and IHC are shown in Figure 4.

**Table 2:** Immunohistochemical findings in 16 pediatric IMT patients.

Case	ALK	SMA	Vimentin	Desmin	CD117	S-100	Dog-1	Ki-67
1	+	+	+	Partially+	−	−	NP	5%
2	−	+	+	−	NP	−	NP	10%
3	+	+	+	NP	NP	−	NP	10%
4	+	+	−	−	−	−	NP	3%
5	+	−	+	+	NP	−	NP	5%
6	+	+	NP	NP	NP	−	−	10%
7	−	+	+	+	−	−	−	20%
8	+	NP	+	+	NP	−	NP	5%
9	+	+	−	−	−	−	NP	2%
10	−	+	+	NP	−	−	−	10%
11	+	+	NP	+	weakly+	−	−	35%
12	−	+	−	+	−	−	−	5%
13	+	Partially+	+	+	−	−	−	5%
14	−	Partially+	+	+	−	−	−	5%
15	−	+	+	NP	−	−	−	10%
16	+	+	+	NP	−	−	NP	10%

NP, not performance.

### 3.5 Treatment and survival outcomes

All 16 pediatric patients underwent surgical treatment as first-line therapy. Fifteen procedures achieved clear (R0) margins; one subglottic lesion was margin-positive (R1) after endoscopic resection. No adjuvant radiotherapy or chemotherapy was administered after primary surgery. The treatment strategies and follow-up information for all pediatric patients are summarized in Table 1.

With a median follow-up of 41 months (Range: 12–108), three patients (18.8%) developed local recurrence and underwent repeat resection. Their clinicopathological details are summarized below.

Case 10: A 4-year-old boy underwent primary en-bloc resection of a mesenteric IMT (8.5 cm × 7.8 cm × 5.5 cm) with regional lymph-node dissection. Margins were negative (R0) and all 12 lymph nodes were tumour-free. No adjuvant therapy was given. Eleven months later he developed colicky abdominal pain, nausea and bilious vomiting. Contrast-enhanced CT revealed a 6.0 cm heterogeneous, partially necrotic mass adherent to small-bowel loops with upstream dilatation, consistent with recurrent IMT and low-grade obstruction. Emergency laparotomy confirmed the diagnosis; segmental small-bowel resection with primary anastomosis achieved a second R0 resection. The child has remained disease-free for 54 months after the second operation.

Case 11: A 9-year-old boy presented with a 9.8 cm × 8.0 cm × 7.2 cm mesenteric IMT that was completely excised with clear margins. No adjuvant treatment was administered. Nineteen months post-operatively he developed progressive abdominal distension and weight loss. Imaging demonstrated a 7.0 cm cystic-solid mass in the right lower quadrant and multiple peritoneal nodules. Second-look laparotomy with right hemicolectomy and peritoneal debulking confirmed multifocal IMT. Given peritoneal dissemination, adjuvant crizotinib was initiated. At 46 months the patient remains alive with stable, partially responding disease on imaging.

Case 16: A 7-year-old girl with subglottic IMT presented with critical inspiratory stridor. Diagnostic angiography revealed a hypervascular lesion; embolisation and conservative airway management failed to relieve the obstruction. He underwent tracheotomy followed by endoscopic excision; final margins were focally positive (R1). Three months later, fibre-optic laryngoscopy showed a 1 cm granulation-type recurrence causing 30% luminal narrowing. Endoscopic holmium-laser ablation achieved complete endoscopic clearance. The airway remains patent and the patient is disease-free 48 months after the second procedure.

By June 2025, 15 patients are alive without evidence of disease; one patient (Case 11) is alive with controlled peritoneal disease on ALK inhibition. No treatment-related mortality or distant metastasis was observed.

## 4 Discussion

IMT is a rare mesenchymal neoplasm of intermediate malignant potential, histologically comprising bland spindle-shaped myofibroblasts within a variable chronic inflammatory milieu (1, 9). Before being formally designated as IMT, it was referred to by various terms, including inflammatory pseudotumor, plasma cell granuloma, fibrous xanthoma, myxoid hamartoma, and benign myofibroblastic tumor, among others (10). Despite shared histology, IMT exhibits unpredictable biology: most pursue an indolent course, yet intra-abdominal and pediatric lesions display higher rates of local recurrence and, rarely, metastasis (3, 11). Precise etiology remains elusive; proposed triggers include antecedent trauma, infection, prior therapy, or germline predisposition, all potentially inciting uncontrolled myofibroblast proliferation (12–14). The present study seeks to delineate clinicopathologic correlates and outcome determinants in pediatric IMT, where such nuances directly inform risk-adapted management.

Pediatric IMT is typically diagnosed within the first two decades of life (15). In our cohort, ages ranged from 4 months to 15 years (median 5.0 years), with a modest male preponderance (M:F = 1.7:1) that most likely reflects limited sample size rather than a true sex bias. The disease can occur in various anatomical locations, but the abdominal and pelvic organs are the most common sites, followed by the head and neck, lungs, trunk, and limbs (3, 6, 16). Consistent with prior reports, abdominal and pelvic locations were most frequent in our cohort, often presenting with nonspecific gastrointestinal symptoms, including abdominal distension, pain, vomiting, fever, and palpable abdominal masses (17, 18). Head and neck lesions typically manifested as slow-growing masses (15, 19), whereas pulmonary involvement mimicked chronic infection (8, 20). Additionally, systemic inflammatory manifestations—fever, weight loss and laboratory abnormalities—are reported in 15%–30% of children with IMT (21, 22). Our findings corroborate this pattern: on admission, 43.8% of patients had leukocytosis, 56.3% had anaemia, 41.7% showed elevated CRP and 60.0% had raised ESR, whereas conventional tumour markers remained within normal limits. Importantly, these inflammatory indices-and their associated constitutional symptoms—reverted to normal within three months of complete tumour resection. This rapid postoperative normalization supports their use as simple, cost-effective surrogate markers for early detection of recurrence in settings where molecular surveillance is unavailable.

Ultrasound remains the first-line modality in children because it is rapid, non-invasive and does not require sedation (23). In the present cohort it consistently demonstrated hypoechoic solid masses with either circumscribed or infiltrative margins and scant internal vascularity, findings that were concordant with the mesenteric IMT series described by Qian et al. (23). Nevertheless, these features overlap with those of other pediatric soft-tissue neoplasms, and none of our lesions could be confidently classified pre-operatively.

In the imaging evaluation of pediatric IMT, both CT and MRI offer distinct advantages, particularly MRI, which provides superior clarity in demonstrating the relationship between the tumor and surrounding soft tissues, thereby offering valuable information for treatment planning and prognostic assessment (24). On contrast-enhanced CT the tumours were heterogeneously attenuated, with variable patterns of enhancement (peripheral, progressive, or delayed) and occasional central necrosis or punctate calcification, as previously reported (24). On MRI scans, most lesions appear as soft tissue masses, with T1-weighted imaging (T1WI) showing isointense or hypointense signals and T2-weighted imaging (T2WI) showing isointense or hyperintense signals. The signal characteristics are closely related to the degree of fibrosis and inflammatory cell infiltration within the lesion. Contrast-enhanced MRI demonstrates a variety of enhancement patterns (24, 25). However, neither modality permitted reliable differentiation from malignant sarcoma or lymphoma.

Endoscopic evaluation (laryngoscopy, bronchoscopy or gastrointestinal endoscopy) was invaluable for submucosal lesions; in two cases it revealed smooth polypoid masses that were subsequently excised endoscopically (26). 18F-FDG PET-CT was not routinely employed, reflecting its recognized limitation in IMT: variable tracer uptake related to cellularity and inflammation frequently results in false-positive results (27). Previous studies have reported cases where IMT was misdiagnosed as lymphoma on PET-CT (28). While PET-CT cannot reliably diagnose IMT due to its variable FDG uptake, it remains a valuable tool for detecting primary tumors, assessing local recurrence and distant metastasis, as well as evaluating treatment response (29).

In our study, ultrasound was performed in 13 children, revealing hypoechoic solid masses with well-defined margins. Particularly in cases of abdominal IMT, ultrasound effectively diagnosed complications such as intestinal intussusception and bowel obstruction. However, it remained challenging to definitively characterize the nature of the lesions preoperatively. Nine patients underwent CT scans, and two patients underwent MRI scans, but the imaging findings lacked specificity and failed to provide a correct preoperative diagnosis. This highlights that pediatric IMTs exhibit diverse imaging features, some of which may mimic malignancy. However, relying solely on preoperative imaging for a definitive diagnosis remains difficult.

Currently, pathological examination and IHC remain the “gold standard” for diagnosing IMT, with ALK gene rearrangement and ALK protein expression serving as crucial diagnostic criteria (9, 30). Microscopically, the tumour is composed of cytologically bland, spindle-shaped myofibroblasts arranged in fascicles, accompanied by a dense mixed inflammatory infiltrate rich in lymphocytes, plasma cells and eosinophils (31). IHC plays a critical role in the diagnosis of IMT by identifying the immunophenotype of myofibroblasts and excluding other similar diseases (30). In IMT, SMA and vimentin are typically positiven (32), desmin may be focally expressed, whereas S-100, CD34 and CD117 are consistently negative (31, 33). ALK is particularly significant for the diagnosis of IMT due to its relatively high sensitivity and specificity. It is also recognized as one of the driver genes of IMT (34). ALK immunoreactivity was detected in 62.5% (10/16) of our cases; SMA, vimentin and desmin positivity were observed in 80.0% (12/15), 78.6% (11/14) and 63.7% (7/11), respectively.

Owing to insurance constraints, FISH for ALK rearrangement was performed in only three patients, yielding two positive and one negative result, consistent with prior series reporting ALK rearrangements in 50%–70% of cases (33). The ALK gene may fuse with multiple partner genes, such as TPM3/4, GCC2, TRAF3, EML4, and THBS1, which could be one of the key mechanisms underlying the development of IMT (35–37). In ALK-negative tumours, alternative drivers have been identified, including ROS1, RET, NTRK3 and PDGFRB rearrangements or mutations (32, 38, 39). These alterations define a genomically distinct subset that may respond to corresponding TKIs (e.g., entrectinib for NTRK3, selpercatinib for RET, avapritinib for PDGFRB) (39, 40). Although our cohort was not systematically profiled for these drivers, we acknowledge that prospective protocols should incorporate comprehensive molecular panels to capture actionable fusions and to refine risk-adapted therapy in ALK-negative cases.

Although malignant transformation is reported in 8%–18% of IMTs, characterised by loss of spindle morphology, increased mitoses and necrosis (41), none of our three recurrent specimens displayed evidence of high-grade progression. This may reflect the indolent biology of pediatric lesions, early detection of relapse, or the small number of recurrences studied. Serial sampling of recurrent or metastatic foci is therefore warranted to capture any evolution toward aggressive disease (42, 43).

Pre-operative diagnosis can be challenging. Image-guided core-needle or endoscopic biopsy is often attempted for deep-seated lesions, but limited material, crush artefacts and dense inflammation frequently yield inconclusive results (44). In a recent pediatric series, initial biopsies failed to establish the diagnosis in 3 of 4 IMT cases, mandating repeat sampling or upfront resection (45). ALK-negative or IgG4-mimicking tumours are particularly prone to misclassification. Thus, when clinical-radiological suspicion remains high despite a non-diagnostic biopsy, clinicians should consider i. larger-gauge or incisional re-biopsy, ii. frozen-section-guided early excision, or iii. direct definitive surgery if morbidity is acceptable.

Contemporary guidelines establish complete surgical excision with histologically negative margins as the cornerstone of therapy for pediatric IMT (22, 46). The operative strategy is dictated by tumour site, size and local infiltration, with the primary objective of achieving R0 resection whenever anatomically feasible (9). Published series indicate that children undergoing R0 resection experience excellent long-term outcomes, with 5-year survival consistently exceeding 90% (47). Adjuvant treatment after R0 resection remains contentious. Current evidence does not support the routine addition of radiotherapy or chemotherapy in this setting, especially in view of the potential long-term sequelae of irradiation—namely growth retardation and secondary malignancy—which have led to recommendations against radiotherapy in children under three years (48). For patients with unresectable disease, incomplete excision (R1/R2) or documented recurrence, systemic options are considered (15). Corticosteroids, non-steroidal anti-inflammatory drugs and cytotoxic regimens containing anthracyclines with vincristine or vinblastine have produced objective response rates of 48%–64%, with durable disease control reported in selected cases (48, 49).

Beyond histopathology, molecular profiling now guides therapeutic decisions. ALK rearrangements are detected in more than half of pediatric IMTs, providing a rational target for precision therapy (40, 50). In advanced, unresectable or relapsed ALK-positive disease, ALK inhibition has moved from salvage to adjuvant strategy.Ceritinib achieved an objective response rate of 70% among ten heavily pre-treated children (51), while crizotinib produced complete remission in seven of fourteen pediatric patients and partial remission in five, for an overall response rate of 86% (52). These data establish ALK-directed therapy as an effective first-line option in genomically defined subsets. ALK-negative tumours lack this target and may pursue a more aggressive course, with higher metastatic risk. Empirical approaches—including NSAIDs, rituximab and alternative tyrosine-kinase inhibitors—have shown anecdotal benefit in this subgroup (53, 54), but prospective validation is required to define optimal management.

With increasing understanding of pediatric IMT, neoadjuvant therapy has gradually gained attention for its application in pediatric IMT and has demonstrated significant clinical benefits in some patients (55). Pre-operative ALK inhibition or chemotherapy can down-stage initially unresectable tumours, enabling R0 resection while maximally preserving adjacent organs (50). In a minority of cases, this approach has induced complete remission, thereby obviating the need for extensive surgery (56). Consequently, neoadjuvant therapy should be considered for lesions in anatomically challenging sites or for patients with early recurrence, although prospective protocols are required to define optimal timing, duration and patient selection.

Pulmonary IMT deserves particular mention because it represents the most frequently reported visceral site in both children and adults. According to two recently published studies, lung IMTs account for the majority of thoracic cases and are characterized by a distinctly indolent biology: complete surgical resection is associated with a recurrence rate as low as 2%, whereas incomplete resection carries up to a 60% recurrence risk (57, 58). The rarity of metastatic disease (<5%) and the excellent long-term survival after R0 resection support the current recommendation that surgery remains the cornerstone of treatment, with systemic therapy reserved for unresectable or relapsed disease (58). These data corroborate our own observation that the single pulmonary IMT in our cohort (Table 1, Case 8) has remained disease-free for 46 months after complete resection and reinforce the importance of achieving negative margins irrespective of anatomic site.

Pediatric IMT generally has a favorable prognosis, yet the recurrence rate varies with the anatomical location of the tumor. It has been reported that the recurrence rate for pulmonary IMT is approximately 2%–5%, while for extra-pulmonary IMT, the recurrence rate is significantly higher, around 25% (15, 59). Distant metastasis is relatively rare in pediatric IMT. Multivariate analyses have identified tumour location, local invasiveness, multi-focal growth, positive surgical margins, ALK expression status and specific gene fusions as independent risk factors (9, 31, 60). In particular, mesenteric or peritoneal disease with a multinodular pattern, as well as tumors situated in the pharynx, larynx or sinonasal tract, recur more frequently, presumably because complete surgical extirpation is technically challenging (61, 62). Across studies, margin status emerges as the single most robust predictor of local relapse and ultimately influences both survival and subsequent recurrence risk (48, 59, 63).

Optimal therapy for recurrent pediatric IMT has not been prospectively defined. In fit children with limited local relapse, repeat resection remains the treatment of choice, provided complete excision is achievable (49). In our cohort, all 16 patients underwent primary surgery (1 endoscopic, 15 open); 15 obtained R0 margins and none received adjuvant therapy. During a median follow-up of 41 months, three patients (18.8%) developed local recurrence and underwent second operations. Two underwent repeat R0 resections without further therapy. The third, who presented with multi-focal peritoneal disease, achieved disease stabilisation on crizotinib after incomplete debulking. Notably, the index resections of all three recurrences shared several features: one originated from an R1 endoscopic excision, and the two mesenteric primaries measured 8 cm and 10 cm, respectively. Additionally, the Ki-67 labeling index was markedly higher in recurrent lesions (mean 18.3%) than in non-recurrent tumors (8.5%), although this observation is limited by the small sample size.These data align with recent adult and pediatric series suggesting that Ki-67 > 10% may identify a subset with increased proliferative activity and a higher likelihood of local relapse (59, 64). While Ki-67 is not yet a validated prognostic biomarker in IMT, it could complement tumour site, margin status and molecular subtype in future multi-parametric risk-stratification models.

## 5 Conclusions

Pediatric IMT is a rare, low-grade neoplasm with an excellent prognosis after complete surgical excision. Pre-operative diagnosis remains challenging because of non-specific clinical and radiological features; histopathology and a targeted IHC panel remain indispensable. R0 resection is the cornerstone of therapy; adjuvant treatment, if required, should be individualised according to tumour site, margin status, Ki-67 index and other clinicopathological risk factors. Given the potential for recurrence and metastasis in pediatric IMT, long-term monitoring and regular follow-up are crucial for early detection of recurrence or metastatic lesions.

## Data Availability

The original contributions presented in the study are included in the article/Supplementary Material, further inquiries can be directed to the corresponding author.
